# MAPK signaling pathway contributes to regulating the Nrf2 expression and compensation for hypoxia in hypoglossal nucleus induced by chronic intermittent hypoxia in rats

**DOI:** 10.3389/fneur.2026.1742054

**Published:** 2026-03-30

**Authors:** Rui Cao, Jing Wang, Renjing Ye, Zhuoding Luo, Ya Wen Shi, Min Yin

**Affiliations:** The First Affiliated Hospital of Nanjing Medical University, Nanjing, China

**Keywords:** chronic intermittent hypoxia (CIH), hypoglossal nucleus, mitogen-activated protein kinase (MAPK), Nrf2/Keap1 pathway, obstructive sleep apnea syndrome (OSA)

## Abstract

**Introduction:**

To investigate the expression of classic markers of oxidative stress and the possible triggers, the expression of Nrf2, Keap1, and MAPK pathway-related proteins (JNK, p38MAPK, ERK) were evaluated in the hypoglossal nucleus under a rat model of chronic intermittent hypoxia (CIH).

**Methods:**

A total of 18 adult male Sprague-Dawley rats were randomly divided into 3 groups, including 2 CIH groups (3 weeks and 8 weeks, respectively) and a control group. The CIH groups were fed in low oxygen cabins, in which the fraction of oxygen volume circulated between 5% and 21%, while the control group was fed in atmospheric environment cabins. After 3 and 8 weeks, the expressions of Keap1, Nrf2, JNK, p38MAPK, and ERK in the hypoglossal nucleus of each group were observed by immunohistochemistry.

**Results:**

Immunohistochemical analysis showed that Nrf2 expression in the CIH groups increased as the duration of hypoxia was prolonged. However, the Keap1 expression remained the same after 3 and 8 weeks of CIH. The expressions of JNK, p38MAPK, and ERK in the hypoglossal nucleus of the CIH groups were higher than those of the control group. The expressions of JNK, p38MAPK, and ERK in the CIH-8w group were higher than those of the CIH-3w group. The expressions of p-JNK and p-p38MAPK in the ventral nucleus were significantly higher than those in the dorsal nucleus in the CIH groups.

**Discussion:**

MAPK pathway is activated by CIH and may lead to the accumulation of Nrf2 in the hypoglossal nucleus. The different reactions of the ventral and dorsal nucleus to CIH may relate to their regulating effect on the upper airway muscle groups.

## Introduction

Obstructive sleep apnea (OSA) is a disease characterized by chronic intermittent hypoxia (CIH) of the body and tissues due to the repeated collapse of the upper airway, affecting over 1 billion people worldwide (1). This process could subsequently lead to a series of reactions including systemic inflammation, sympathetic excitation and oxidative stress (2–4). These CIH-induced reactions could be observed in all systems including the central nerve system (CNS), which plays an important role in the contribution of neuronal apoptosis in the cerebral cortex, cerebellum and brainstem (5–7). On the other hand, the response could also include regulatory feedback and protection against hypoxia. Our previous work revealed that the dorsal and ventral nuclei of the hypoglossal nucleus showed different responses to CIH, with overexpression of 5-hydroxytryptamine (5-HT) and 5-hydroxytryptamine Receptor 2A (5-HT 2A) receptors in the ventral nucleus (8). Herein, based on accumulating evidence, we proposed that the increased expression of 5-HT receptors in CIH could suggest a modulation of the airway and protection against of hypoxia by the hypoglossal nucleus.

Many signaling pathways are involved in the CIH-induced injury and the relevant compensation including the mitogen-activated protein kinase (MAPK) pathway, which is regulated by 5-HT (9, 10). The p38MAPK pathway was reported to be activated after hypoglossal nerve crush injury, which in turn led to the apoptosis of neurons in the hypoglossal nucleus (11). However, whether the p38MAPK pathway in the hypoglossal nucleus contributes to the compensation of airway muscle remains unknown.

In mammalian cells, there are three well-defined subgroups of MAPKS. The MAPK family includes three main subfamilies: extracellular signal-regulated kinase (ERK), c-Jun N-terminal kinase (JNK), and p38 MAPK. MAPKs are activated by stimulation such as hypoxia and enter the nucleus to regulate transcriptional regulation, and are involved in the growth, development, apoptosis of cells as well as the recognition, transmission and amplification of intercellular biochemical signals (12, 13). Therefore, in this study, we will investigate the response of MAPK pathways under different levels of CIH and the possible role of MAPK pathways in the compensation of CIH.

## Materials and methods

### Animals

A total of 18 healthy male Sprague–Dawley (SD) rats (8-weeks, clean-grade), weighing between 180 and 200 g, were provided by the Animal Experimental Center of Jiangsu Province. The SD rats were randomly divided into 3 groups, with 6 rats in each group (*n* = 6 per group). The rats in the two CIH groups were fed in an intermittent hypoxia environment for 3 weeks (the CIH 3w group) and 8 weeks (the CIH 8w group) respectively, whereas those in the control group were fed in a normoxic environment. The CIH groups were placed in a chamber with oxygen concentration ranging from 5 to 21% for 2 min in each cycle. Each cycle consists of 1 min of nitrogen filling and 1 min of air filling to maintain the oxygen concentration fluctuating between 5 and 21% in the chamber. The control group was housed in a normoxic chamber with an oxygen concentration of 21%. The CIH group was placed in a CIH environment only from 8:30 to 16:30 daily and returned to the normoxic environment at the end of daily modeling. Except this, all groups were fed and watered freely and kept in the same environment and condition. The rats were all housed in the SPF environment at the Nanjing Medical University Animal Center. The experimental protocol (I ACUC-1901047) was approved by the Experimental Animal Ethics Committee of Nanjing Medical University.

### Immunohistochemistry

Anesthetized animals were euthanized and fixed with 4% paraformaldehyde (pH 7.4, 4 °C) and the medulla oblongata was removed afterward. Having placed in 4% paraformaldehyde in a 4-degree refrigerator overnight (no more than 24 h), the removed medulla oblongata was then dehydrated, placed in 75% alcohol for 1.5 h, 95% alcohol for 1.5 h, 95% alcohol for 1 h, anhydrous ethanol 1.5 h, anhydrous ethanol 1 h, Xylene I 0.5 h, Xylene II 0.5 h.

After completion of dehydration, pathology experiments were performed. The dehydrated brainstem was embedded with paraffin and cut into 4-μm-thick cross-sections in a microtome, starting at a distance of 13.3 mm from bregma according to the Paxinos and Watson atlas. In all 15 brain slices. Among them, slices 1, 6 and 11 were used for Kelch-like ECH-associated protein 1 (Keap1) immunohistochemical staining, slices 2, 7 and 12 were used for nuclear factor erythroid 2-related factor 2 (Nrf2), slices 3, 8 and 13 were used for JNK, brain slices 4, 9 and 14 were used for p38MAPK, and brain slices 5, 10, and 15 were used for ERK. To reduce experimental error, all brain slices were hydrated and repaired with thermal antigens immediately after being dewaxed at room temperature. The primary antibody was incubated at 4 °C for 24 h, and replaced with the same dilution ratio of PBS as a negative control. After the incubation, the sections were rewarmed at room temperature for 30 min and washed three times with PBS. Then, 50 μL of secondary antibody was added dropwise and incubated for 10 min at room temperature. 100 μL of DAB solution was added dropwise after three washes of PBS, the positive material was observed to be dark brown under the microscope. Finally, the average optical density value was analyzed by the IPP Image analysis system (average optical density value = cumulative optical density/area).

### Statistical processing

Data were presented as mean ± standard deviation. The results of immunohistochemistry were performed by one-way analysis of variance (ANOVA) followed by Bonferroni’s *post hoc* test using SPSS 18.0 software (IBM Corp., Armonk, NY, United States). Statistical significance was considered with *p*-value less than 0.05 (*p* < 0.05).

## Results

### Immunohistochemical staining of Keap1 and Nrf2

**Figure 1:**
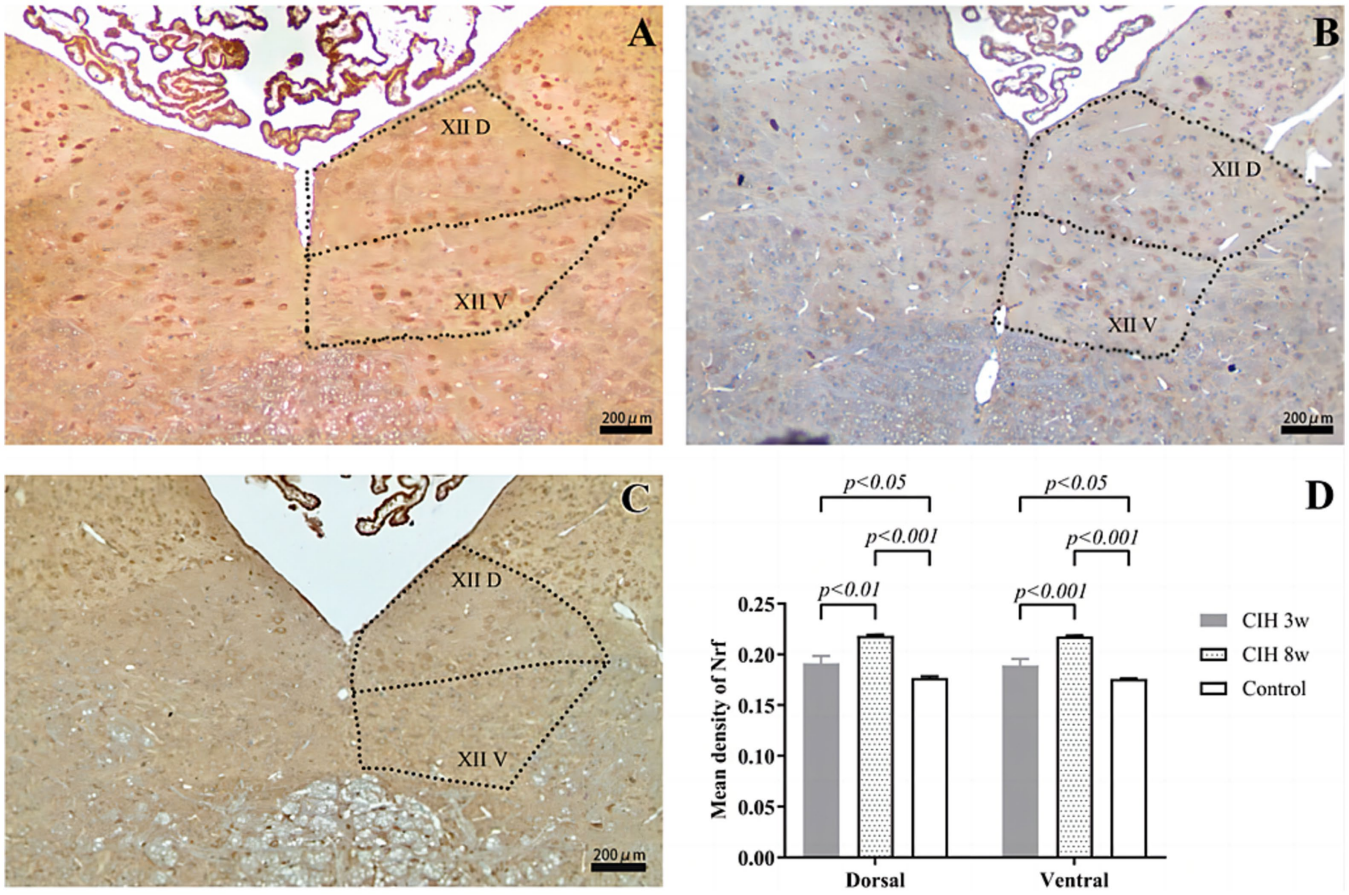
The effect of CIH stimulation on Nrf2 expression was investigated by an immunofluorescence study in CIH 3w **(A)**, CIH 8w **(B)**, and control **(C)**. The mean optical densities of different groups and different nuclei were compared **(D)**. Statistical significance was defined as *p* < 0.05; *p* < 0.01; *p* < 0.0001. Magnification: 4 × objective.

Compared with that in the control group, the mean optical density of Nrf2 in the CIH groups showed a significant increase (Figure 1) as the duration of hypoxia was prolonged. Taking the dorsal nucleus as an example, compared to the control group, the average optical density of Nrf2 was higher in the CIH 3w (*p* < 0.05) and CIH 8w (*p* < 0.001). Meanwhile, the expression of p-JNK is higher in the CIH 8w group than in the CIH 3w group (*p* < 0.01).

**Figure 2:**
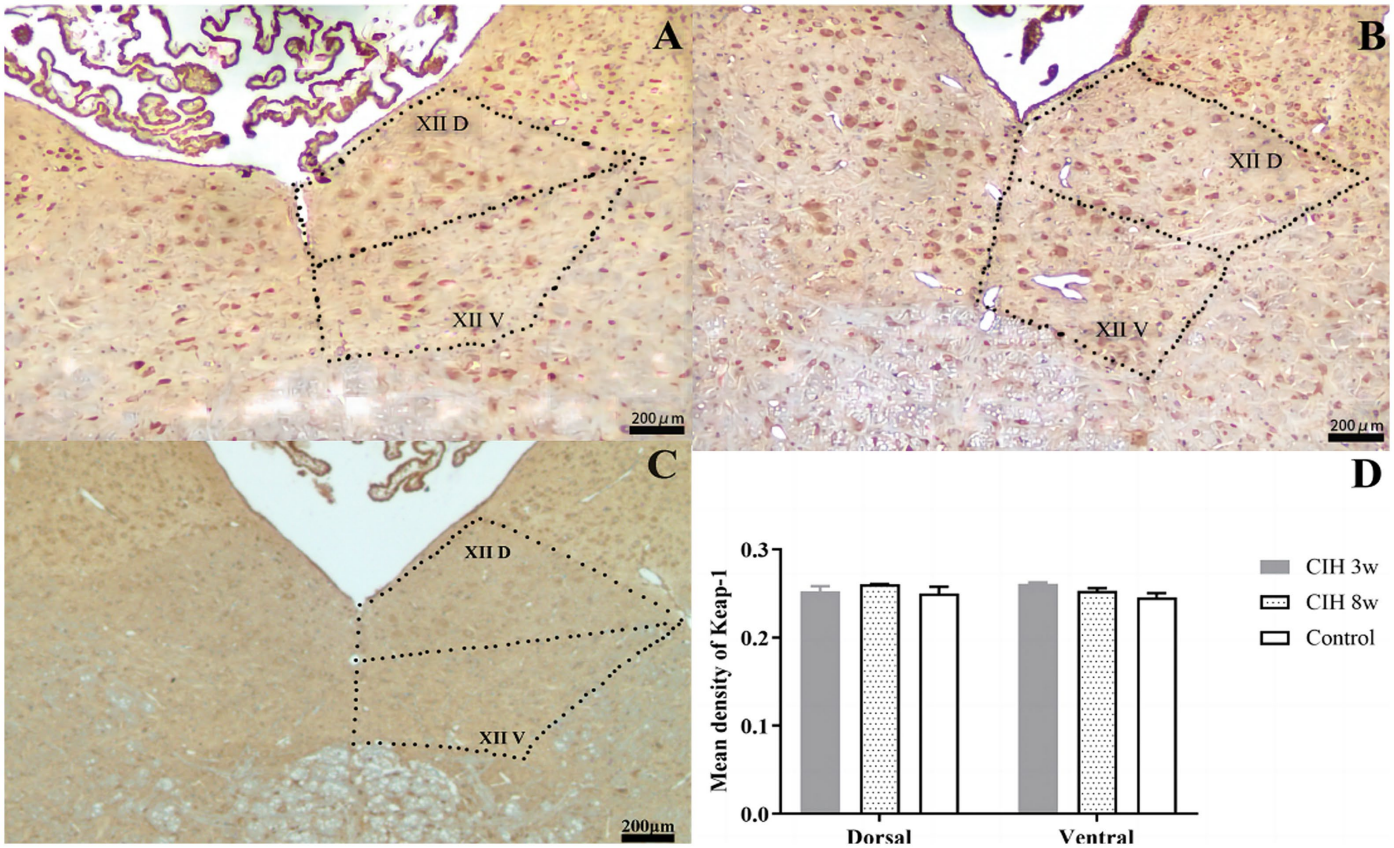
The effect of CIH stimulation on Keap-1 expression was investigated by an immunofluorescence study in CIH 3w **(A)**, CIH 8w **(B)**, and control **(C)**. The mean optical densities of different groups and different nuclei were compared **(D)**. There is no significant differences after 3 W and 8 W of CIH condition. XIID: Dorsal nucleus of hypoglossal nucleus; XIIV: Ventral nucleus hypoglossal nucleus. Magnification: 4 × objective.

However, the Keap1 expression showed no significant differences after 3 W and 8 W of CIH condition (Figure 2). Both Keap1 and Nrf2 expression showed no significant difference between the ventral and the dorsal nuclei at any timepoint.

### Immunohistochemical staining of p-ERK

**Figure 3:**
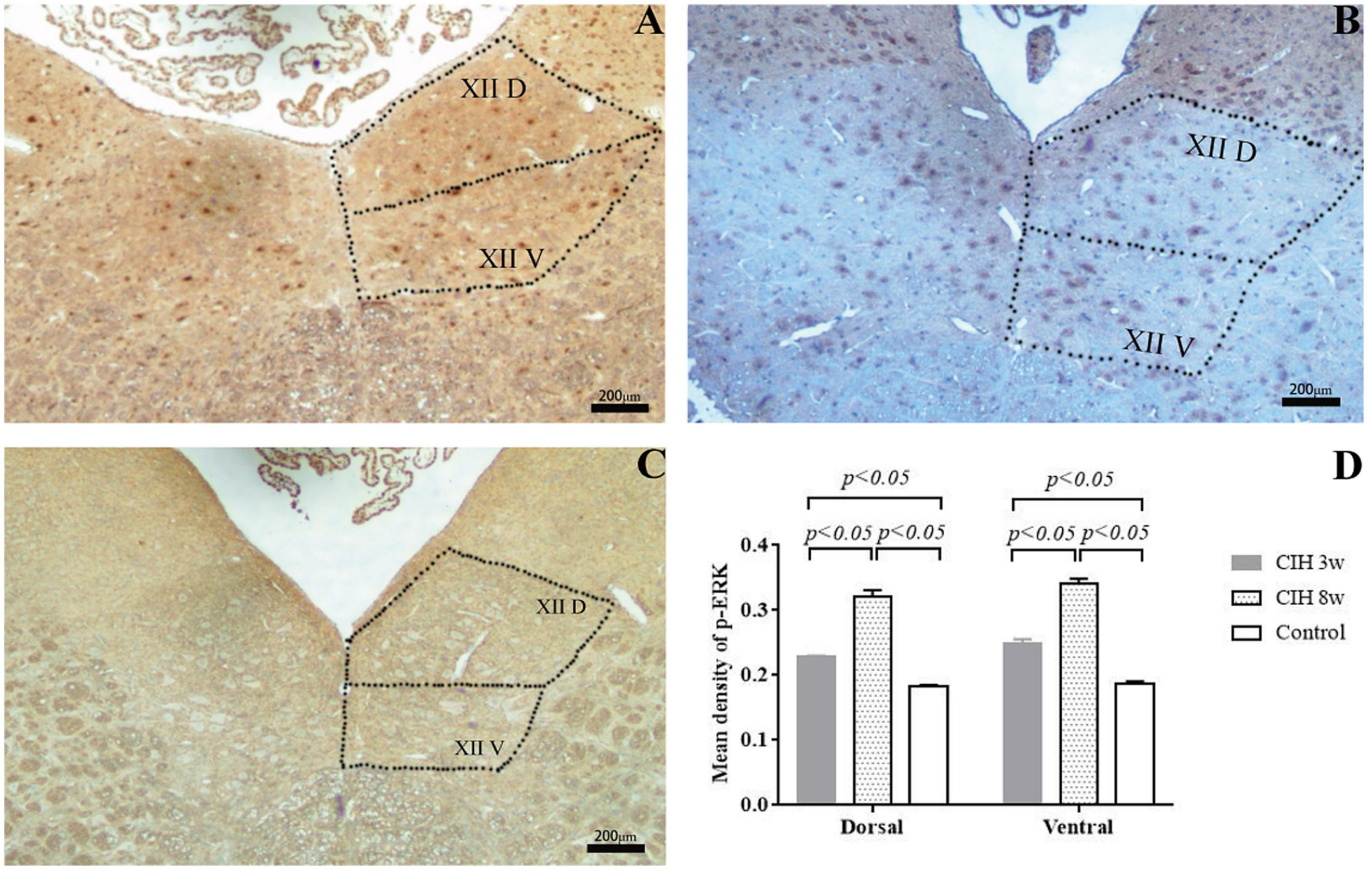
The expression of p-ERK in the ventral and the dorsal nuclei of the hypoglossal nucleus. The p-ERK expressions of CIH 3w **(A)**, CIH 8w **(B)**, and control **(C)** were evaluated by immunofluorescence study. The mean optical densities of p-ERK in the CIH groups were evaluated and compared **(D)**. One-way analysis of variance (ANOVA) was used for comparison between groups. Statistical significance was defined as *p* < 0.05. Magnification: 4 × objective.

The expression of p-ERK was expressed in both ventral and dorsal nuclei of the hypoglossal nucleus. Compared with that in the control group, the mean optical density of p-ERK in the CIH groups showed a significant increase (Figure 3). This trend was obvious in the CIH 3w group (*p* < 0.05) and further increased in the CIH 8w group (*p* < 0.05). However, in the CIH 3w and the CIH 8w group, p-ERK expression showed no significant difference between the ventral and the dorsal nuclei.

### Immunohistochemical staining of p-JNK

**Figure 4:**
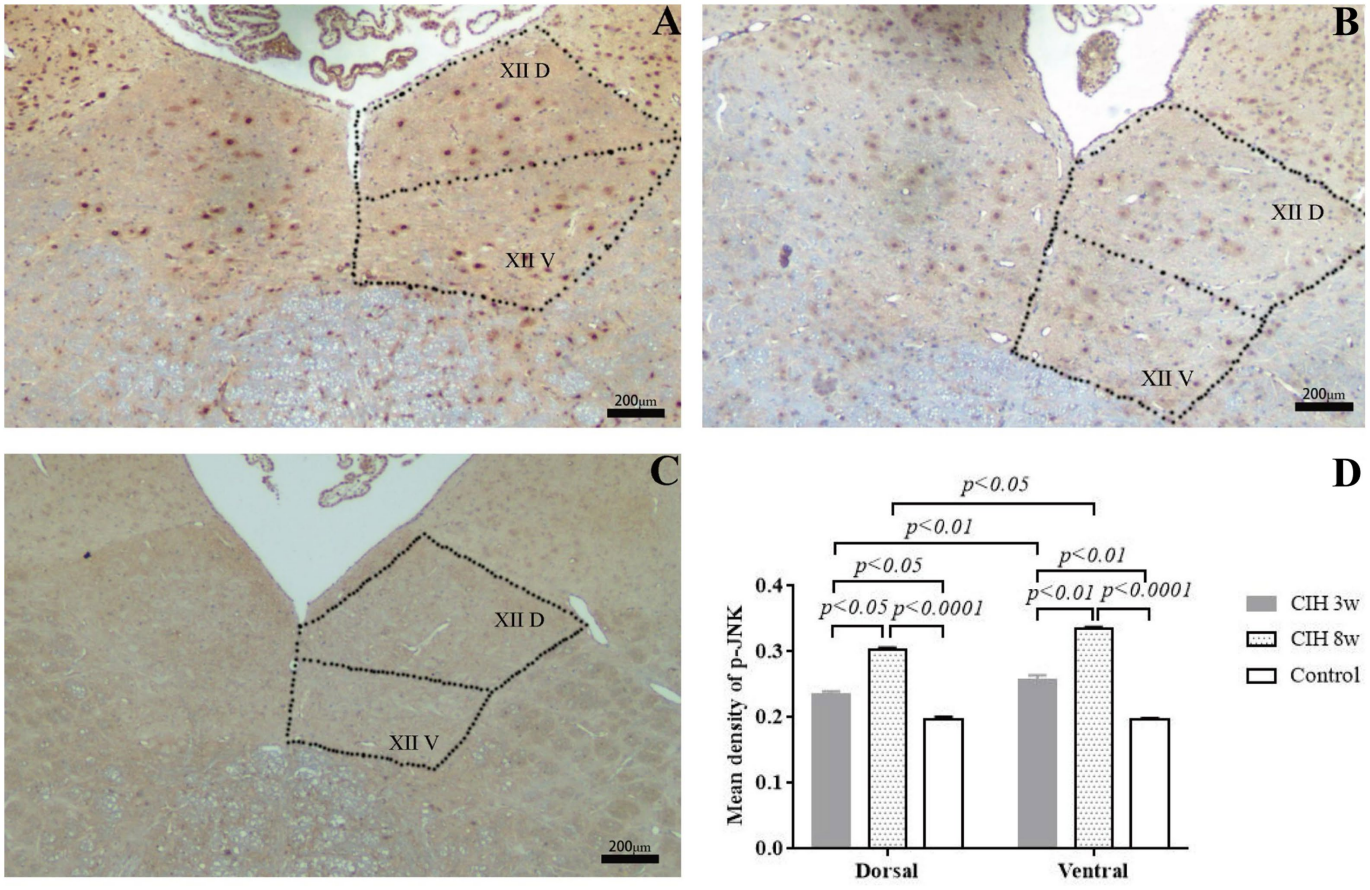
The effect of CIH stimulation on p-JNK expression was investigated by an immunofluorescence study in CIH 3w **(A)**, CIH 8w **(B)**, and control **(C)**. The mean optical densities of different groups and different nuclei were compared **(D)**. Statistical significance was defined as *p* < 0.05; *p* < 0.01; *p* < 0.0001. Magnification: 4 × objective.

P-JNK was also expressed in the ventral and dorsal nuclei of the hypoglossal nucleus, but represented different responses to CIH stimulation, with significantly higher expression in the ventral nucleus (Figure 4). In the CIH 3w group, compared to the dorsal nuclei, the expression of p-JNK in the ventral nucleus is higher (*p* < 0.01). And in the CIH 8w group, there was also a consistent trend (*p* < 0.05). The average optical density of p-JNK was also significantly enhanced by CIH stimulation, showing an increasing trend from 3 to 8 weeks. Taking the dorsal nucleus as an example, compared to the control group, the average optical density of p-JNK was higher in the CIH 3w (*p* < 0.0001) and CIH 8w (*p* < 0.0001). Meanwhile, the expression of p-JNK is higher in the CIH 8w group than in the CIH 3w group (*p* < 0.01).

### Immunohistochemical staining of p-p38MAPK

**Figure 5:**
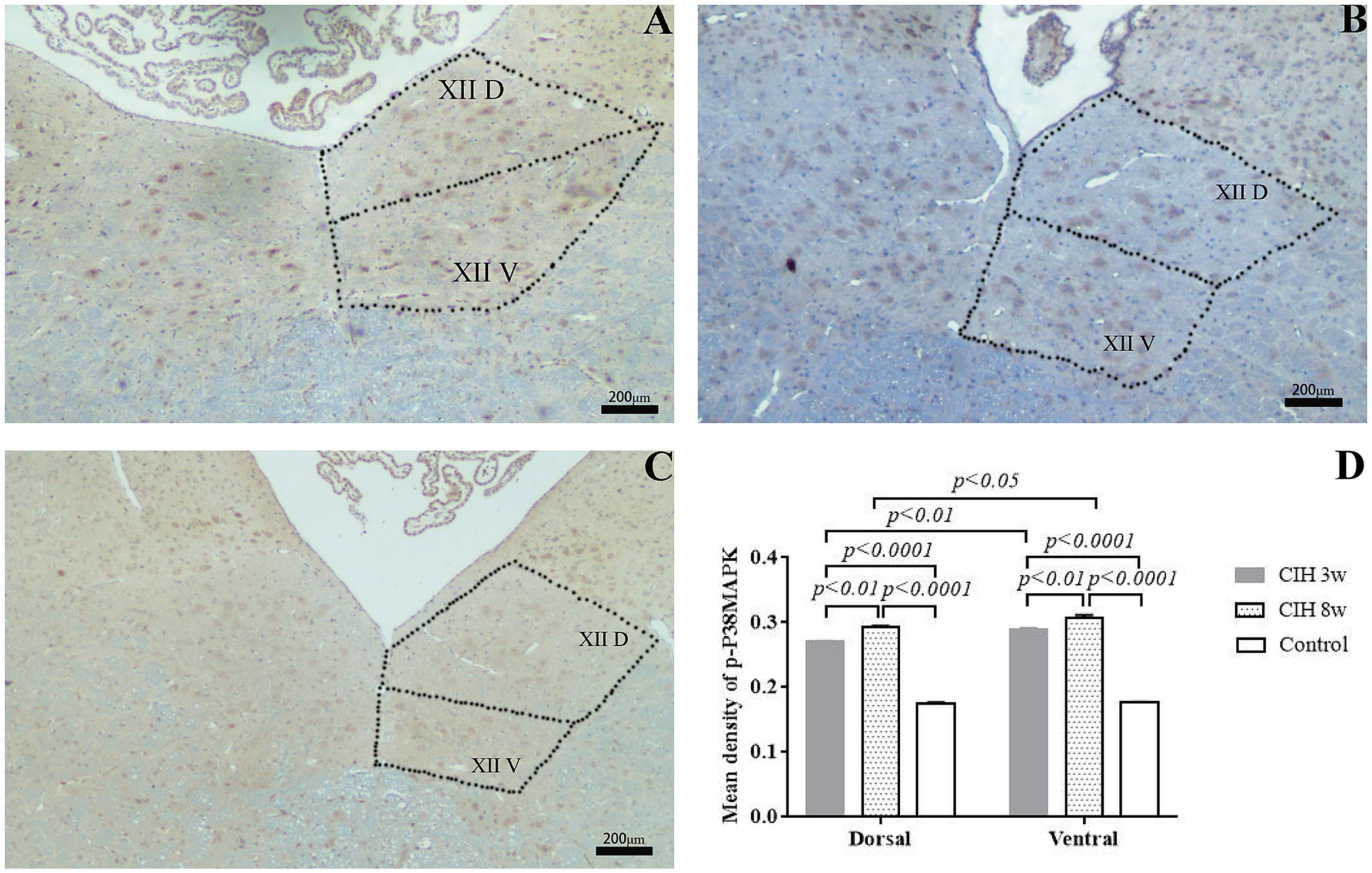
The effect of CIH stimulation on p-p38MAPK expression was investigated by immunofluorescence in CIH 3w **(A)**, CIH 8w **(B)**, and control **(C)**. The mean optical densities of different groups and different nuclei were compared as well **(D)**. Statistical significance was defined as *p* < 0.05; *p* < 0.01; *p* < 0.0001.

The expression trend of p-p38MAPK in the hypoglossal nucleus was consistent with that of p-JNK (Figure 5). The expression of p-p38MAPK was significantly increased in the ventral and the dorsal nuclei, and was more expressed in the ventral nucleus. In the CIH 3w group, compared to the dorsal nuclei, the expression of p-p38MAPK in the ventral nucleus is higher (*p* < 0.01). And in the CIH 8w group, there was also a consistent trend (*p* < 0.05). Besides, the average optical density of p-p38MAPK in the CIH group showed an enhanced trend with CIH duration. Taking the dorsal nucleus as an example, compared to the control group, the average optical density of p-JNK was higher in the CIH 3w (*p* < 0.0001) and CIH 8w (*p* < 0.0001). Meanwhile, the expression of p-JNK is higher in the CIH 8w group than in the CIH 3w group (*p* < 0.01).

## Discussion

CIH is a prolonged process involving recurrent hypoxia/reoxygenation (H/R) condition. Under the H/R condition, the disrupted oxidation–reduction in many organs and tissues can add to the local oxidative stress and lead to cellular damage (14, 15). In our study, we confirmed the existence of increased oxidative stress in hypoglossal nuclei by finding the altered expression of Keap1 and Nrf2, two key factors of the Keap1/Nrf22 signaling pathway. Previous studies have revealed that the Keap1/Nrf22 signaling pathway participates in oxidative stress injury through cell autophagy and ferroptosis (16, 17). Nrf22, which is believed to have a critical protective effect against the cellular damage caused by oxidative stress, was found upregulated in our study. Unlike the factors in the MAPK signaling pathway, the upregulation of Nrf2 showed no difference between the ventral and dorsal parts of hypoglossal nuclei, indicating that the increased expression of Nrf2 may represent the self-defense response of the neurons in the hypoglossal nucleus to oxidative stress injury under CIH conditions, rather than the regulation of tongue muscle movement. The expression of Keap1, which is believed to be a negative regulator of Nrf22, was not found decreased in our study though, making us wonder if there existed other triggers for the activation of Nrf22.

One previous study proposed a regulation of Nrf22 by the MAPK signaling pathway in response to oxidative stress (18). This regulation was proved to be independent of the Nrf22 protein stability controlled by Keap1, but related to the phosphorylation of Nrf22 by p-MAPKs (19). Therefore, we further investigated the altered phosphorylation of MAPK signaling pathways in CIH, including the p-JNK, p-p38MAPK and p-ERK.

Our findings suggested three trends. Firstly, compared with the control group, the CIH groups presented an increased expression of p-JNK, p-p38MAPK and p-ERK in the hypoglossal nucleus. Secondly, the expressions of p-JNK, p-p38MAPK and p-ERK increased along with the prolonged CIH duration, which was demonstrated by the higher expressions of these three factors in the hypoglossal nucleus in the CIH 8w group than in the CIH 3w group. Thirdly, the ventral nucleus had higher expressions of p-JNK and p-p38MAPK than the dorsal nucleus. The increase in phosphorylated MAPKs may be one of the driving factors for the accumulation of Nrf2 in hypoglossal nuclei, eventually inducing the anti-oxidant effect in neurons.

In addition to a possible role in mitigating neuronal oxidative stress damage, the phosphorylation and activation of MAPKs may contribute to the compensation of CIH as well. CIH has been previously reported to activate MAPKs in other tissues, including arteries, pancreas and liver, etc. (20–23). However, only a few studies focused on the mediating role of MAPKs in brainstem nuclei (24–26). 5-HT, a classic neurotransmitter, can regulate spinal respiratory motor plasticity by phrenic long-term facilitation through the MAPK signaling pathway in hypoxia condition (27). Some studies identified the role of 5-HT on the hypoglossal nucleus in CIH as well, indicating the possible activation of the MAPK pathway by 5-HT (10, 28).

The ventral and dorsal nuclei of the hypoglossal nucleus regulate the open and closed airway muscle groups respectively, and respond inconsistently to CIH. The dorsal and the ventral side of the hypoglossal motor nucleus showed different responses to CIH in rats. 5-HT is related to the increased movement of the tongue, indicating its role in compensation of CIH (29). However, the exact mechanism of 5-HT-related compensation in CIH remained unclear while our research solved this problem. In our study, the MAPK/JNK/ERK pathway was significantly activated in the hypoglossal nucleus. The degrees of activation differed between the dorsal and ventral parts of the hypoglossal nucleus, which we believed could be the result of the inconsistent expression of 5-HT and 5-HT-2A receptors. In our previously published work, the expression of 5-HT and 5-HT-2A receptors was found to vary in the dorsal and ventral parts of the hypoglossal nucleus, and the ventral expression of 5-HT and 5-HT-2A receptors was significantly higher than that in dorsal, which not only consists with but also perfects the previous study (8, 30). In this study, p-JNK and p38MAPK showed a similar pattern with activation of 5-HT receptors in the hypoglossal nucleus, supporting the possibility that the ventral nucleus of the hypoglossal nucleus controls the open airway muscle groups. Therefore, we have sufficient evidence to believe the activation of 5-HT and the relevant phosphorylation of MAPK/JNK/ERK will compensate the CIH.

Besides, the activation of the MAPK/JNK/ERK pathway also was enhanced with the prolonged duration of intermittent hypoxia, which may be regulated by nuclei of the telencephalon and diencephalon. Several studies have reported the altered volume, connectivity and metabolic level of nuclei in the telencephalon and diencephalon of OSA patients with longer illness (31–33). The altered nuclei include the hypothalamus and amygdala, which are found targeting the hypoglossal nucleus (34). These nuclei which targeted the hypoglossal nucleus were also able to transmit glutamatergic signaling for arousal in OSA, indicating their roles in stimulating the hypoglossal nucleus in the long term (35). The stimuli from the higher level of the neural system are likely to regulate the hypoglossal nucleus and therefore the airway muscles through the altered expression of 5-HT. One of the evidence is that in sudden unexpected death in epilepsy, respiratory depression by impairment of 5-HT neuromodulation is believed to be the main cause (36). Therefore, it is reasonable to believe that the activation of the MAPK/JNK/ERK pathway in the hypoglossal nucleus is due to the 5-HT neuromodulation from the higher level of CNS in the context of CIH.

The activation of MAPK may be involved in both adaptive and non-adaptive (or harmful) processes, and its functional outcome is determined by the intensity, duration, and specific cellular context. In summary, our study revealed the accumulation of Nrf2 and the potential existence of oxidant stress injury in hypoglossal nuclei of rats in CIH. Then we further investigated the possible trigger for Nrf2 upregulation, which was the activation of the MAPK signaling pathway. We also discussed other effects of hypoglossal nuclei on airway muscles in compensation of CIH that may be delivered by the increased MAPK phosphorylation. This study is limited to a morphological-molecular analysis. Therefore, subsequent studies are required to further investigate the musculature controlled by the hypoglossal nerve. To some degree, our work provides new evidence to the research in CIH and hypoglossal nuclei. However, there are still plenty of mysteries in CIH including to what extent the accumulation of Nrf2 and compensation of upper airway muscles is induced by MAPKs, which remain to be solved in the future.

## Conclusion

In this study, we initially found the accumulation of Nrf2 and discussed the possible triggers. Then we further investigated the site-specific trends of MAPK signaling pathway expression in the hypoglossal nucleus under CIH, which suggested potential damage and compensation mechanisms of the hypoglossal nucleus under CIH. In future work, we will further explore the compensatory functions of the hypoglossal nucleus and MAPK signaling pathways on the innervated muscle groups in the context of CIH.

## Data Availability

The original contributions presented in the study are included in the article/supplementary material, further inquiries can be directed to the corresponding author.
